# FISH-negative *BCR::ABL1-*positive e19a2 chronic myeloid leukaemia: the most cryptic of insertions

**DOI:** 10.1186/s12920-023-01607-7

**Published:** 2023-07-26

**Authors:** Philippa C. May, Alistair G. Reid, Mark E. Robinson, Jamshid S. Khorashad, Dragana Milojkovic, Simone Claudiani, Genomics England Research Consortium, Fenella Willis, Jane F. Apperley, Andrew J. Innes

**Affiliations:** 1 Centre for Haematology, Department of Immunology and Inflammation, Imperial College London, London, UK; 2 Specialist Integrated Haematological Malignancy Service, Great Ormond Street Hospital for Children, NHS Foundation Trust, London, UK; 3 North West Genomic Laboratory Hub, Manchester NHS Foundation Trust, Manchester, UK; 4 Center of Molecular and Cellular Oncology, Yale Cancer Center, Yale School of Medicine, New Haven, CT USA; 5 Clinical Genomics, The Centre for Molecular Pathology, The Royal Marsden NHS Foundation Trust, London, UK; 6 Department of Clinical Haematology, Imperial College Healthcare NHS Trust, London, UK; 7 Department of Haematology, St George’s University NHS Foundation Trust, London, UK

**Keywords:** Cryptic, Myeloid, Fusion gene, False negative, *BCR:ABL1*

## Abstract

**Background:**

Chronic myeloid leukaemia (CML) is one of the most well characterised human malignancies. Most patients have a cytogenetically visible translocation between chromosomes 9 and 22 which generates the pathognomonic *BCR::ABL1* fusion gene. The derivative chromosome 22 (‘Philadelphia’ or Ph chromosome) usually harbours the fusion gene encoding a constitutively active *ABL1* kinase domain.

A small subset of patients have no visible translocation. Historically, these ‘Philadelphia chromosome negative’ patients caused diagnostic confusion between CML and other myeloproliferative neoplasms; it is now well established that the *BCR::ABL1* fusion gene can be generated via submicroscopic intrachromosomal insertion of *ABL1* sequence into *BCR*, or, more rarely, of *BCR* into *ABL1*. The fusion genes arising from cryptic insertions are not detectable via G-banded chromosome analysis [karyotype] but can nevertheless always be detected using fluorescence in situ hybridisation (FISH) and/or qualitative reverse transcriptase PCR.

**Case presentation:**

A 43-year-old female presented with suspected CML in 2007; however, contemporaneous gold standard laboratory investigations, G-banded chromosome analysis and FISH, were both negative. The reverse transcriptase quantitative PCR (RT-qPCR) assay available at the time, which was capable of detecting the common *BCR::ABL1* transcripts (e13a2/e14a2), was also negative. Upon review in 2009, the newly recommended reverse transcriptase multiplex PCR (capable of detecting all *BCR::ABL1* transcripts including the atypical ones) subsequently detected an e19a2 fusion. The patient then responded to tyrosine kinase inhibitor therapy. In contrast, FISH studies of both samples with three commercially available probes remained consistently negative.

Retrospective whole genome sequencing, undertaken as part of the 100,000 Genomes Project, has now revealed that the patient’s *BCR::ABL1* fusion gene arose via a uniquely small insertion of 122 kb *ABL1* sequences into *BCR*.

**Conclusions:**

We present a patient with suspected chronic myeloid leukaemia whose genetic investigations were originally negative at the time of diagnosis despite the use of contemporaneous gold standard methods.

This is the first report of a FISH-negative, *BCR::ABL1* positive CML which demonstrates that, even after sixty years of research into one of the most well understood human malignancies, whole genome sequencing can yield novel diagnostic findings in CML.

## Background

CML is one of the most well characterised human malignancies; the toponymous Philadelphia chromosome was discovered in 1960 using some of the earliest genetic techniques [1]. Using newly developed G-banded chromosome analysis in 1973, the Philadelphia chromosome was then established to be a derivative chromosome arising from a balanced translocation between chromosomes 9 and 22 [2]. The involvement of *BCR* and *ABL1* in the fusion gene was established in 1984 and 1986, respectively [3, 4]. This early knowledge of the *BCR::ABL1* fusion gene has resulted in CML being the target of many laboratory and clinical firsts, including bone marrow transplantation [5], international standardisation of molecular monitoring [6, 7] and one of the first targeted cancer therapies, imatinib, a tyrosine kinase inhibitor [8].

The *BCR::ABL1* fusion gene can be found in four main variant forms (major, minor, micro and nano) defined by the breakpoint location within the *BCR* gene, and thus the size of the resulting fusion protein. Whilst the breakpoints in *ABL1* are relatively tightly clustered in a region surrounding exons 1b to 2, breakpoints in *BCR* can occur within any of several relatively large regions of the gene. Those falling within the major breakpoint cluster region (M-bcr) yield the common 210 kDa fusion protein (P210^*BCR::ABL1*^) which is highly specific for chronic myeloid leukaemia [3]. The M-bcr region spans *BCR* exons 12 to 16 and is approximately 4 Kb. Philadelphia-positive acute lymphoblastic leukaemia (ALL) is also defined by the *BCR::ABL1* fusion gene, however, the majority of fusion genes in ALL arise from rearrangements within the minor breakpoint cluster region (m-bcr). This 72 kb m-bcr region between *BCR* exons 1 and 2 gives rise to the P190^*BCR::ABL1*^ protein [9], found in CML only rarely [10]. The 2 kb micro breakpoint cluster region (µ-bcr) spans *BCR* introns 18 to 22; these rare transcripts were not identified until more recently [11] and have been associated with neutrophilia [12] and a less severe disease course. Lastly, a handful of patients have been reported with a breakpoint in the nano (ν-bcr) breakpoint cluster region around exon 6 [13].

Approximately 1–2% of CML patients have an apparently normal karyotype. In these patients, the *BCR::ABL1* fusion gene arises as a consequence of an insertion of *ABL1* sequences into *BCR,* or vice versa [14]. Such submicroscopic rearrangements are termed ‘masked Ph’ or cryptic insertions. Although it can be inferred that cryptic insertions arise from a minimum of three breakage events, as opposed to the two required for a standard translocation, the presence of a simple, balanced cryptic insertion does not appear to have a prognostic impact [15].

Whilst undetectable by karyotype analysis, cryptic insertions can be detected using FISH [16], reverse transcriptase multiplex PCR and/or the appropriate RT-qPCR assay for the transcript type. Hence, the European Leukaemia Net guidelines in 2013 [17] *recommended* multiplex PCR at diagnosis to identify the fusion transcript type, and the 2020 guidelines now state that this is *mandatory* [18]. This recommendation was introduced based on the need to avoid a false impression that a patient with a rare transcript is in complete molecular response after TKI treatment, if tested with the incorrect RT-qPCR assay.

## Case presentation

**Fig. 1:**
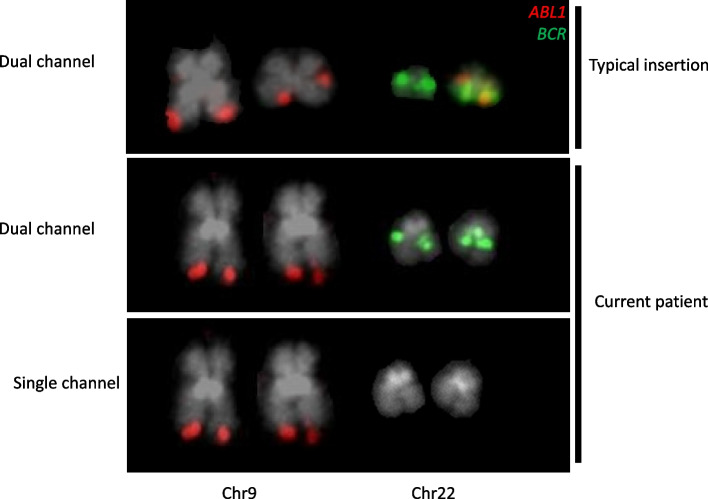
fluorescence in situ hybridisation using dual colour dual fusion probe for *BCR* (green) and *ABL1* (red) showing (top) dual channel image of a derivative chromosome 22 arising from a standard cryptic insertion, (middle) dual channel image of the current patient’s derivative chromosome 22 and (bottom) as above, showing only the single Texas Rad channel. No green signal is detectable

A 43-year old female presented with thrombocytosis of 1453 × 10^9^/L and a mild leucocytosis of 23.2 × 10^9^/L, with left shifted myelopoiesis and neutrophils of 16 × 10^9^/L, eosinophils of 1 × 10^9^/L and basophils of 0.4 × 10^9^/L suspicious of CML. However laboratory investigations performed according to the contemporaneous best practice guidelines [19] did not support this: G-banded chromosome analysis showed a normal female karyotype in all metaphases examined (46,XX[20]) and FISH for *BCR::ABL1* was negative (see Fig. 1). Her marrow was hypercellular with increased megakaryocytes and reticulin. On account of the clinical picture and absence of a BCR::ABL1 fusion, she was diagnosed with a Philadelphia-negative myeloproliferative neoplasm and treated accordingly with hydroxycarbamide.

**Fig. 2:**
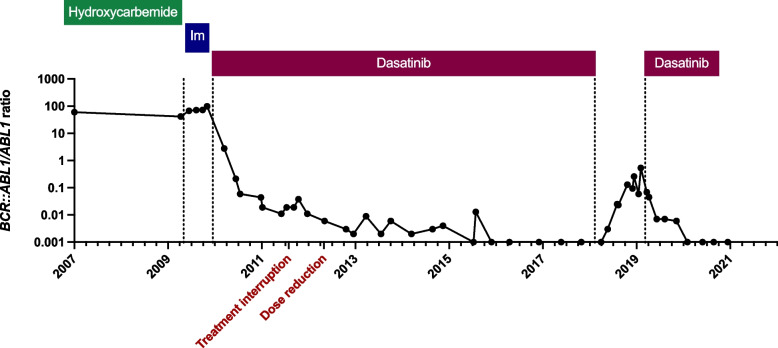
Plot showing the patient’s *BCR::ABL1/ABL1* ratio since presentation; vertical dotted lines indicate treatment changes; Im, imatinib

Upon the development of progressive basophilia, characteristic of CML, laboratory investigations were repeated two years later. The newly introduced qualitative PCR, designed to identify all known *BCR:ABL1* transcripts [13], detected an e19a2 (µ-bcr) fusion transcript. Retrospective re-examination of cDNA from her first sample confirmed this transcript had been present, however, re-examination of fixed cell suspensions from the same two dates using three different commercially available FISH probes were consistently negative. There were no robust reports of FISH negative CML but a revised diagnosis of CML was made based on the qualitative PCR results. The patient was started on imatinib and achieved complete haematological response. The cryptic nature of the *BCR::ABL1* fusion made cytogenetic monitoring impossible, but there was no molecular response. She was subsequently changed to a second generation TKI, dasatinib, and achieved a deep molecular response, with eventually undetectable *BCR::ABL1* by RT-qPCR. Treatment free remission was attempted in 2018, but this resulted in loss of molecular response, and dasatinib was restarted. She subsequently re-achieved and has maintained a deep molecular response to date (Fig. 2).

**Fig. 3:**
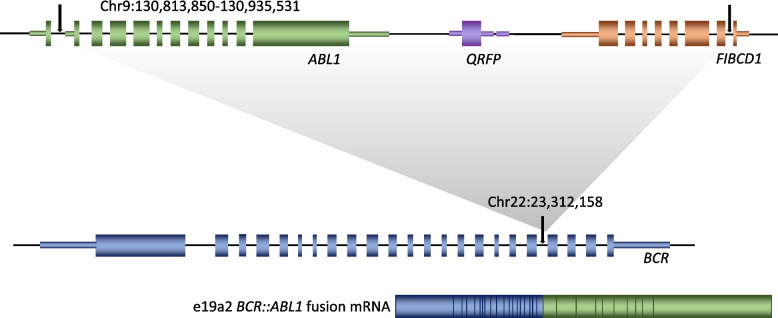
Schematic showing the chromosome 9 sequence (top) inserted into chromosome 22 *BCR* (middle) and the resulting e19a2 *BCR::ABL1* fusion transcript (bottom)

Recently whole genome sequencing (WGS) of DNA derived from the patient’s diagnostic peripheral blood sample, and undertaken as part of the 100,000 Genomes Project, has revealed a 121,681 bp insertion of *ABL1* sequence (chr9:130,813,850–130,935,531) into intron 19 of *BCR* at chr22:23,312,158 (all coordinates refer to ENST00000318560.6/NM_005157.6 and GRCh38; see Fig. 3). This is, to our knowledge, the smallest reported *ABL1* insertion giving rise to a functional *BCR::ABL1* fusion protein in a CML patient.

## Discussion and conclusions

Cryptic insertions of *ABL1* sequences into *BCR,* or vice versa*,* are not unusual. Approximately 1–2% of CML patients have no apparent Ph chromosome; undertaking FISH in suspected CML referrals with a normal karyotype has thus long been routine. When qualitative reverse transcriptase PCR testing at diagnosis of CML was first recommended by European LeukemiaNet in 2013, this was principally to determine the appropriate transcript for subsequent monitoring in order to avoid falsely assuming a patient had achieved a complete molecular response. This case is unique in that the patient presented with a rare e19a2 fusion and a uniquely small submicroscopic insertion.

**Fig. 4:**
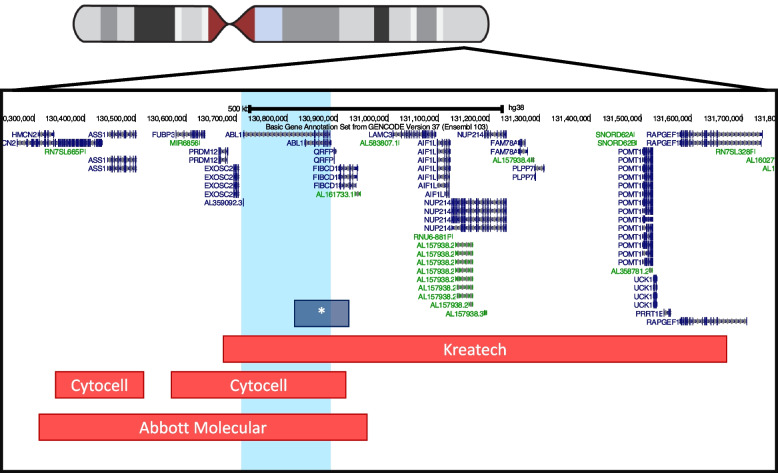
Schematic showing region of interest from UCSC genome browser with GENCODE v37 transcripts indicated. *ABL1* is highlighted in light blue and the size of the current patient’s cryptic insertion is indicated with an asterisk (*). Approximate locations (according to package inserts) of commercial FISH probes are indicated with red bars

The insertion was not detectable using any of three commercially available probes at the time (Abbott Molecular, Illinois; Kreatech Diagnostics, Amsterdam; and Cytocell, Cambridge) despite all three manufacturers’ probe maps indicating they had full coverage of the entire *ABL1* coding region and at least 300 kb of sequence up or downstream (Fig. 4). Analysis included dual and single channel examination of at least 200 interphases and/or metaphases, single channel inspection of both fluorochromes and digital enhancement (i.e. artificially boosting the signal to enable visualisation) of both fluorochromes; no ectopic signals could be identified contemporaneously or retrospectively Fig. 1. Until recently, FISH probes were manufactured by nick translation labelling of bacterial artificial chromosome (BAC) clones, or fragmented subclones thereof. Unfortunately, repetitive sequences can cause background fluorescence and it is thus sometimes necessary to exclude certain clones from the target probe mix, which can lead to gaps in the coverage; this may explain the inability of the three probe systems to detect the *ABL1* insertion seen in this patient, which, though relatively small nevertheless included the majority of the *ABL1* gene and encompassed a region well within the detection limit of FISH. In recognition that FISH probes may have gaps within the probe, FISH probe manufacturers often caveat the probe specification, for example, “breakpoints outside this region, or variant rearrangements wholly contained within this region, may not be detected”. In support of this finding, it is worth noting that a small number of FISH-negative *PML::RARA*-positive cryptic insertions have been reported in acute promyelocytic leukaemia [20]. Unfortunately, due to more than a decade passing between the initial presentation and the whole genome sequencing results, it was not possible to undertake further FISH testing; it is possible that an *ABL1* break apart probe or whole chromosome painting may have sufficiently improved coverage for detection of the insertion.

The 122 kb insertion included almost 40 kb intronic sequence upstream of the second *ABL1* exon (the first exon translated in the *BCR::ABL1* fusion protein) and 50 kb downstream (including an intergenic region, the entire *QFRFP* gene and the majority of the downstream gene *FIBCD1*), all of which are superfluous for the formation of the fusion gene. Hypothetically, an insertion of just 40 kb could contain all the necessary coding sequences for an in-frame *BCR::ABL1* fusion; the smallest possible insertion resulting in a functional *BCR::ABL1* fusion gene could be considerably smaller than the one reported here. Conversely, a reciprocal insertion of *BCR* into *ABL1* would require a minimal insertion of just *BCR* exon 1 to form P190^*BCR::ABL1*^. *BCR*-into-*ABL1* insertions are documented, however all those reported to date have all been large enough to be detected by FISH [21].

In summary, we report the first FISH-negative *BCR::ABL1* positive chronic myeloid leukaemia patient and have been able to fully characterise the fusion. Whole genome sequencing has demonstrated insertion of 122 Kb of coding *ABL1* sequence into intron 19 of *BCR* resulting in a rare in-frame e19a2 fusion transcript, the smallest cryptic insertion reported to date. Despite over 60 years of research into CML and our advanced understanding of its genetics making it the paradigm for the precision medicine era, whole genome sequencing has only now explained a 15-year diagnostic mystery.

## Data Availability

The data that support the findings of this study are available from 100,000 Genomes Project but restrictions apply to the availability of these data, which were used under license for the current study, and so are not publicly available. Data are however available from Genomics England Limited upon reasonable request.

## Abbreviations

ALL: Acute lymphoblastic leukaemia
CML: Chronic myeloid leukaemia
FISH: Fluorescence in situ hybridisation
PCR: Polymerase chain reaction
